# Can-SINE dynamics in the giant panda and three other Caniformia genomes

**DOI:** 10.1186/s13100-018-0137-0

**Published:** 2018-11-10

**Authors:** Changjun Peng, Lili Niu, Jiabo Deng, Jianqiu Yu, Xueyan Zhang, Chuang Zhou, Jinchuan Xing, Jing Li

**Affiliations:** 1 Key Laboratory of Bio-resources and Eco-environment, Ministry of Education, College of Life and Sciences, University of Sichuan, Chengdu, China; 2 Sichuan Wild Animal Research Institute, Chengdu Zoo, Chengdu, China; 3 Sichuan Key Laboratory of Conservation Biology on Endangered Wildlife, College of Life Sciences, Sichuan University, Chengdu, 610065 Sichuan China; 4 Department of Genetics, Human Genetic Institute of New Jersey, Rutgers, The State University of New Jersey, Piscataway, NJ USA

**Keywords:** Caniformia species, Can-SINEs, Transposable element activity, Putative panda-specific elements

## Abstract

**Background:**

Although repeat sequences constitute about 37% of carnivore genomes, the characteristics and distribution of repeat sequences among carnivore genomes have not been fully investigated. Based on the updated Repbase library, we re-annotated transposable elements (TEs) in four Caniformia genomes (giant panda, polar bear, domestic dog, and domestic ferret) and performed a systematic, genome-wide comparison focusing on the Carnivora-specific SINE family, Can-SINEs.

**Results:**

We found the majority of young recently integrated transposable elements are LINEs and SINEs in carnivore genomes. In particular, SINEC1_AMe, SINEC1B_AMe and SINEC_C1 are the top three most abundant Can-SINE subfamilies in the panda and polar bear genomes. Transposition in transposition analysis indicates that SINEC1_AMe and SINEC1B_AMe are the most active subfamilies in the panda and the polar bear genomes. SINEC2A1_CF and SINEC1A_CF subfamilies show a higher retrotransposition activity in the dog genome, and MVB2 subfamily is the most active Can-SINE in the ferret genome. As the giant panda is an endangered icon species, we then focused on the identification of panda specific Can-SINEs. With the panda-associated two-way genome alignments, we identified 250 putative panda-specific (PPS) elements (139 SINEC1_AMes and 111 SINEC1B_AMes) that inserted in the panda genome but were absent at the orthologous regions of the other three genomes. Further investigation of these PPS elements allowed us to identify a new Can-SINE subfamily, the SINEC1_AMe2, which was distinguishable from the current SINEC1_AMe consensus by four non-CpG sites. SINEC1_AMe2 has a high copy number (> 100,000) in the panda and polar bear genomes and the vast majority (> 96%) of the SINEC1_AMe2 elements have divergence rates less than 10% in both genomes.

**Conclusions:**

Our results suggest that Can-SINEs show lineage-specific retransposition activity in the four genomes and have an important impact on the genomic landscape of different Caniformia lineages. Combining these observations with results from the COSEG, Network, and target site duplication analysis, we suggest that SINEC1_AMe2 is a young mobile element subfamily and currently active in both the panda and polar bear genomes.

**Electronic supplementary material:**

The online version of this article (10.1186/s13100-018-0137-0) contains supplementary material, which is available to authorized users.

## Background

Transposable elements (TEs) are mobile DNA sequences that occupy a large proportion of genomes of carnivores [1, 2]. For example, TEs account for about 36.1% of the dog genome [3], 37% of the giant panda genome [4], and 38.1% of the polar bear genome [5]. TEs have a major impact on genomic architecture and can affect genes in many ways, such as gene mutation, gene activation/silencing, mRNA alternative splicing, X-chromosome inactivation, and promoting additional forms of structural genetic variation [6–10]. TEs can generally be divided into class I and class II elements [11, 12]. Class I elements, also called retrotransposons, amplify using a copy-and-paste mechanism via an RNA intermediate [11, 13]. Short interspersed elements (SINEs), long interspersed elements (LINEs) and long terminal repeat (LTR) are different types of retrotransposons. SINE elements have no protein-coding sequences and depend on the enzymatic machinery of LINEs to proliferate [14]. Class II elements, also called DNA transposons, can relocate in the genome using a cut-and-paste mechanism [11].

During evolution, active retrotransposons can proliferate in the genome and accumulate diagnostic nucleotide variations (e.g., substitutions, insertions, and deletions) over time. Based on the diagnostic variations that are shared by all members, retrotransposons have diversified into a variety of subfamilies, each with its own set of diagnostic sequence characteristics and period of activity [15]. In some cases, the relationships of retrotransposon subfamilies indicate hierarchical characteristics, with the youngest subfamilies containing the most diagnostic mutations and oldest subfamilies the least [16]. Understanding proliferation patterns of young active retrotransposons is crucial because the accumulation of new copies and lineage-specific subfamilies in different taxa can contribute to the genome variations between species. For example, a genome-wide comparison of the human and chimpanzee genomes indicated a large number of species-specific L1, *Alu* and SINE-VNTR- *Alu* (SVA) elements that have been inserted since their divergence ~ 6 Million year ago (Mya) [17, 18].

**Fig. 1:**
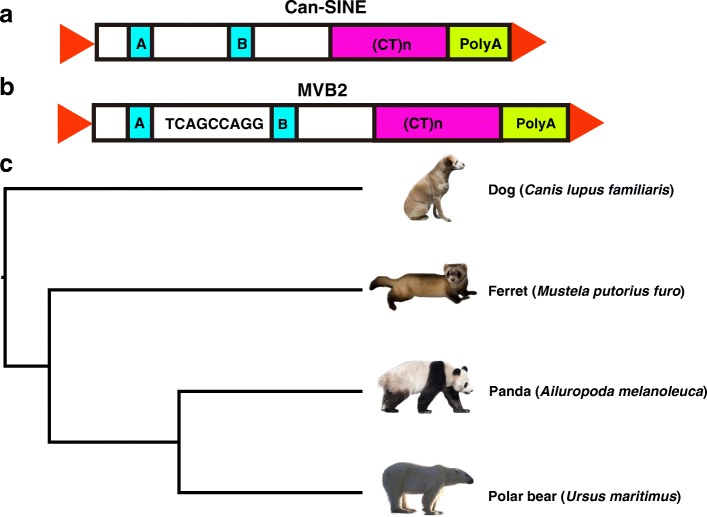
Structure of Can-SINE, MVB2 and the phylogenetic tree of panda, polar bear, dog, and ferret. **a** Can-SINE is normally 150-300 bp long. It is characterized by RNA polymerase III promoter boxes A and B (blue), (CT)_n_ microsatellite (pink) and a ploy(A) tail (yellow). The red triangles represent the 8–15 bp TSD sequence. **b** MVB2 is characterized by a conserved short symmetrical sequence TCAGCCAGG between the two promoter boxes (blue). **c** The phylogenetic relationships of the four Caniformia species were based on TimeTree [63]

Can-SINE, a Carnivora-specific SINE family, was first described in the early 1990s [19]. It is defined by a tRNA-related region, which includes A and B promoter boxes, followed by a (CT)n microsatellite and terminate with a poly-A/T tail containing the polyadenylation signal AATAAA (Fig. 1a) [20]. A typical Can-SINE element is usually 150–300 base pair (bp) in length and flanked by 8–15 bp of target site duplications (TSDs) generated during retrotransposition. Can-SINEs are ubiquitous across Carnivora. Through their continuous activity and accumulation in the genome since the Pholidota-Carnivora split about 59 Mya [21], Can-SINEs have become the predominant TEs and constitute a significant source of genomic variations within Carnivora genomes. The copy number of Can-SINEs is estimated to range from 1.1 [3] to 1.3 [19] million copies based on the dog (*Canis familiaris*) and the harbour seal (*Phoca vitulina concolour*) genomes. Can-SINE elements are divided into different subfamilies, which are named starting with “SINEC” and, followed with species of first discovery (e.g., “Fc” = *Felis catus*, “CF” = *C. familiaris*, and “AMe” = *Ailuropoda melanoleuca*) [22]. The exception is MVB2, a Can-SINE subfamily firstly identified in American mink (*Neovison vison*) genome and named by its “B2” structure that contains regions homologous to the split intragenic RNA polymerase III promoter and a conserved short symmetrical sequence “TCAGCCAGG” between the two promoter boxes [20](Fig. 1b). With the increased number of whole genome sequences of Carnivora species [3, 4, 23, 24], many TE subfamilies are recognized, which greatly enriched Repbase library. In 2011, only 16 Can-SINE consensus sequences described in the Repbase library serve as prototypes for subfamily classification schemes [22], while the number has increased to 25 consensuses by 2015 in Repbase (version 2015.8.7). Characterization of these newly recognized Can-SINE subfamilies in the genomes of different Carnivora species will provide new insight into their impacts on the host genomes.

Caniformia (dog-like carnivores) is a suborder in Carnivora. Species in Caniformia are characterized by significant morphological, ecological, and behavioral variation [25]. In particular, the giant panda is globally iconic due to its physically attractive attributes, almost exclusive bamboo based diet, unique morphological and physiological adaptations, and its characteristic black and white pelage [26]. Analysis of the draft genome of the giant panda published in 2010 indicated at least 70 Mb of TE sequences (3% of genome) have divergence rates ≤10%, and these young elements could contribute to the genomic and functional variations of the species [4]. However, little is known about the transposition activities of young TEs in different Caniformia species and their contributions towards the evolution of Caniformia genomes. In this study, we reannotated TEs and characterized Can-SINEs in the giant panda genome and three other Caniformia species, the domestic dog (*C. lupus familiaris*), polar bear (*Ursus maritimus*), and ferret (*Mustela putorius furo*) using an updated Repbase library. Then, the two-way genome alignments were employed to identify lineage-specific Can-SINE elements, and the putative panda-specific (PPS) elements were identified using the dog, ferret, and polar bear as outgroups (Fig. 1c). These elements integrated into the panda genome recently and were potentially from new Can-SINE subfamilies that are still active in the panda genome. Our study provides new insight into the contributions of Can-SINEs to the genomic diversity in Caniformia species and the important roles of young and active Can-SINEs in shaping the panda genome.

## Results

### TE content in the four Caniformia genomes

Because the TE annotations in the published genomes were generated using different consensus definition, we first reannotated TE content in the four Caniformia genomes using the same Repbase consensus library. The new annotations show that 36.4–39.2% of the four genomes are composed of TEs. The major TE types and their copy numbers are similar in the four genomes (Table 1). LINEs are the most abundant TEs by length and occupy about 20% of the four genomes (19.4–21.8%). SINEs occupy around 9% of the genomes (8.7–10.5%), while they are the most abundant TEs in term of copy number with more than 1.2 million copies in all four genomes. DNA transposons are the least abundant TEs. The comparison of TE divergence rates indicated that the youngest elements in the carnivore genomes are SINEs and LINEs (Additional file 1: Figure S1). The majority of TEs with divergence rates of ≤10% belong to these two groups, whereas most LTR elements and DNA transposons show divergence rates of more than 20%. The number of SINE elements with divergence of ≤10% is about 1.5 times higher in the dog genome than the panda, polar bear, and ferret genomes (Additional file 1: Table S1), implying more active SINE expansion in the dog lineage. However, LINE elements (including segments and full length elements) with divergence of ≤10% is 4 to 5 folds less in the ferret than the other three genomes (Additional file 1: Table S1). Despite the lower level of LINE activity, the young SINE elements in the ferret genome appear to be in line with other genomes. This result might imply that divergence alone is not enough to measure transposition activity.

**Table 1:** Major types of TEs in the panda, polar bear, dog, and ferret genome

	Panda		polar bear		dog		ferret	
Types	Counts	Gp^a^	Counts	Gp	Counts	Gp	Counts	Gp
SINE	1,217,323	8.71%	1,204,559	8.68%	1,572,336	10.50%	1,426,958	9.61%
LINE	980,166	20.90%	962,809	21.77%	917,087	20.84%	912,474	19.42%
LTR	330,037	5.48%	327,472	5.51%	311,552	4.95%	304,753	4.73%
DNA	351,819	3.20%	351,500	3.21%	327,704	2.83%	307,487	2.64%
Total:	2,879,345	38.29%	2,846,340	39.17%	3,128,679	39.12%	2,951,672	36.4%

^a^ *Gp* Genome percentage

### Can-SINE landscape within the four Caniformia genomes

**Fig. 2:**
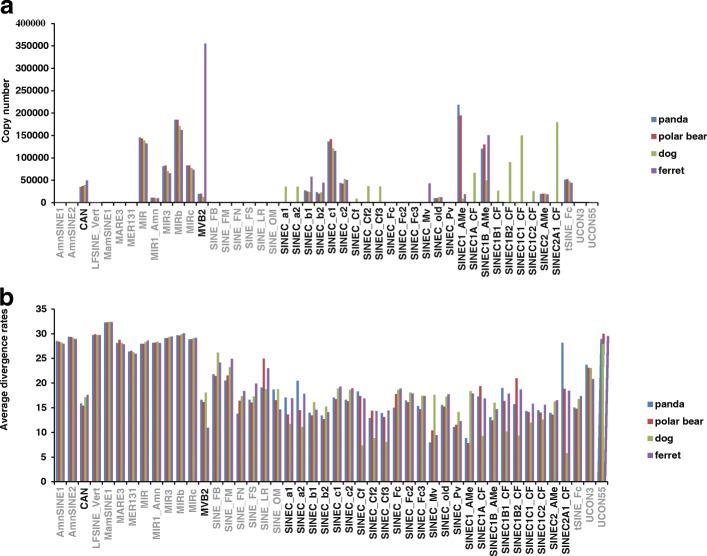
SINE subfamily characteristics in panda, polar bear, dog, and ferret genomes. Copy number (**a**), average divergence rates (**b**) of SINE subfamilies are shown and all Can-SINE subfamilies are labeled in black

Due to the highest copy number and the relative young age of SINEs in the four genomes, we focused the comparative analysis on the SINE elements. A total of 573,625-1,043,529 Can-SINE elements were identified in the four genomes, accounting for 40–66% of all SINEs in each species (Fig. 2a). However, copy numbers and average divergence rates of each Can-SINE subfamilies vary greatly among the four genomes. SINEC1_AMe, SINEC_C1, and SINEC1B_AMe are the three most successful subfamilies and together they account for 75.33% and 75.31% of the total Can-SINEs in the panda and polar bear genomes, respectively. In particular, the copy number of SINEC1_AMe elements in the panda and polar bear genomes are 21.9–24.7 times those in the dog genome, and 10.3–11.5 times those in the ferret genome (Fig. 2a). Whereas nine Can-SINE “CF” subfamilies in the dog genome (SINEC_Cf, SINEC_Cf2, SINEC_Cf3, SINEC1A_CF, SINEC1B1_CF, SINEC1B2_CF, SINEC1C1_CF, SINEC1C2_CF, SINEC2A1_CF) constructe the most abundant super subfamily (more than 600,000 elements), while there are no more than 8000 elements in the other three Caniformia genomes (Fig. 2a).

The average divergence rates of SINE subfamilies indicate that most subfamilies are relatively old (Fig. 2b). The MamSINE1 shows the highest divergence rate (~ 30%) in all four genomes. Only six subfamilies (SINEC_Cf, SINEC_Cf2, SINEC_Cf3, SINEC1A_CF, SINEC1B2_CF, and SINEC2A1_CF) in the dog genome, one subfamily (SINEC_Mv) in the ferret genome, one (SINEC1_AMe) in the polar bear genome, and two (SINEC1_Mv and SINEC1_AMe) in the panda genome have divergence rates of ≤10% (Fig. 2b). SINEC1B_AMe subfamily has the third lowest divergence rate in the panda and polar bear genomes and it has accumulated high copy number in both genomes, suggesting elements in SINEC1B_AMe might have integrated into these genomes recently (Fig. 2b).

### TinT patterns of Can-SINEs in the four genomes

**Fig. 3:**
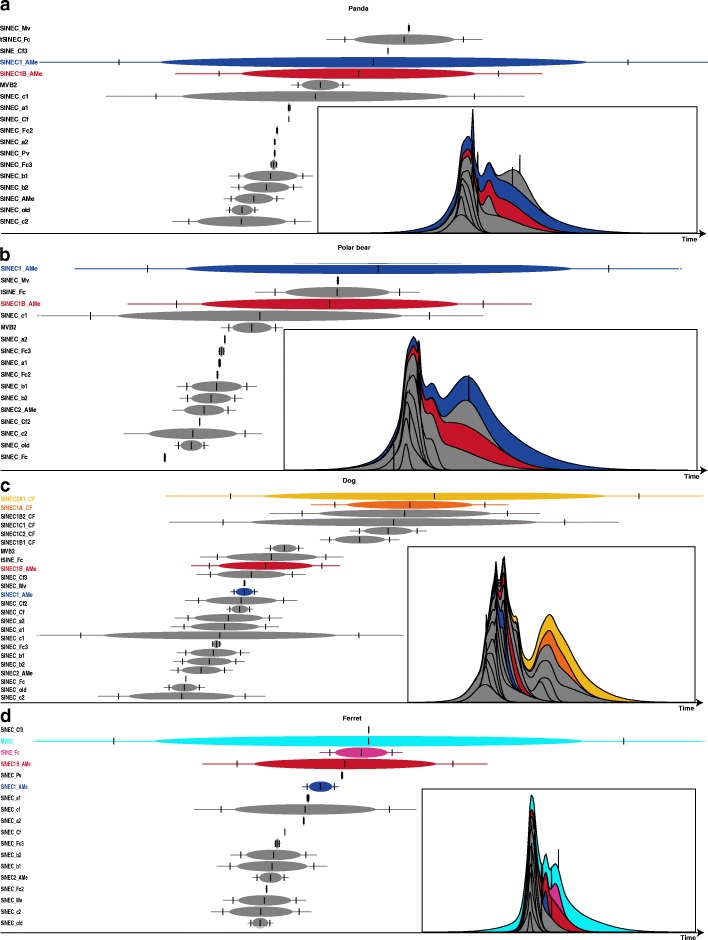
Transposition in transposition (TinT) patterns of SINEs in panda (**a**), polar bear (**b**), dog (**c**), and ferret (**d**) genomes. SINEC1_AMe and SINEC1B_AMe subfamilies are displayed in blue and red in all panels. SINEC2A1_CF andSINEC1C1_CF are displayed in yellow and orange (**c**), MVB2 and tSINE_Fc are displayed in wathet blue and pink (**d**), whereas other SINE subfamilies are colored in grey. A central vertical line indicates the period of maximal activity within the colored ovals. The 75th percentile of the activity range is shown as flanking vertical lines; the terminal lines manifest the 99th percentile range of activity. The timescale runs from left to right is for old to young. The corresponding cumulative likelihoods of element family activity are given as thumbnails in the insets

Based on the principle that old inactive TEs will not insert into young TEs, the transposition in transposition (TinT) analysis can be used to determine the relative age of transposon families [27, 28]. TinT analysis was used to evaluate the relative transposition activities of different Can-SINE subfamilies (Fig. 3). TinT results are consistent with that of the average divergence rates (Fig. 2b). Most subfamilies have short periods of transposition activities. SINEC1_AMe, SINEC1B_AMe, and tSINEC_Fc are the three most recently active Can-SINEs subfamilies in the panda and polar bear genomes that are active for a long period of time. Although some subfamilies such as SINEC_Mv and SINEC_Pv exhibit low divergence rate (Fig. 2b), they are lack of TinT insertions and have small copy numbers (only 612 and 385 SINEC_Mv elements are in the giant panda and the polar bear genomes, respectively). This result suggests that these subfamilies are only active for a short period of time or have low transposition rate (Fig. 3a, b). Unlike panda and polar bear genomes showing similar TinT patterns for Can-SINE subfamilies, TinT patterns in dog and ferret genomes are very different. In the dog genome SINEC2A1_CF and SINEC1C1_CF are the most active subfamilies with the longest activity until recently, whereas SIEC1B_AMe and SINEC1_AMe appear to be less active (Fig. 3c). In the ferret genome MVB2 maintains the highest and longest transposition activity, and SINEC1B_AMe also exhibits relatively high activities compared to other subfamilies (Fig. 3d).

### PPS_SINEC1_AMe and PPS_SINEC1B_AMe elements

**Fig. 4:**
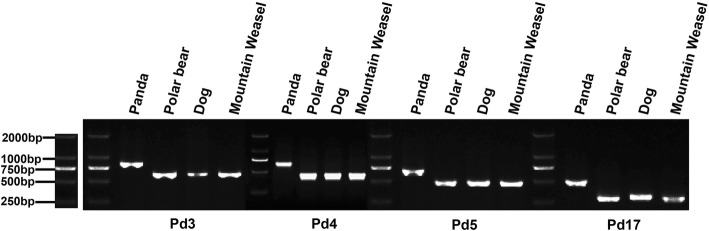
PCR validation of four putative panda-specific SINEs loci (Pd3, Pd4, Pd5 and Pd17) in four carnivore species. An agarose-gel chromatograph of PCR products of four panda specific insertion events. The filled site in Pd3-Pd17 is 850 bp, 638 bp, 686 bp, and 499 bp respectively; and the empty site in Pd3-Pd17 is 637 bp, 439 bp, 477 bp and 281 bp respectively in the dog genome

The two-way genome alignments were used to identify lineage-specific Can-SINEs. We focused on panda specific Can-SINEs because Can-SINE specificity in this species is not well investigated, and they consist of an enormous genomic diversity that may contribute to the unusual characteristics in morphology or physiology of giant panda. The young Can-SINE elements in the panda genome were examined to identify panda-specific Can-SINE elements. Because about 95% of SINEs with average divergence rates of ≤10% are from the SINEC1_AMe and SINEC1B_AMe subfamilies (Additional file 1: Table S1), we focused on these two subfamilies in the following analyses. After filtering for the relatively intact and young elements, we selected 168,340 SINEC1_AMe and 80,346 SINEC1B_AMe elements for the panda-associated two-way genome alignments. Finally, we identified 139 SINEC1_AMe elements and 111 SINEC1B_AMe elements that are present in the panda genome but are absent from the orthologous regions of the other three Caniformia genomes (Additional file 2). To assess the validity of these elements, we randomly selected 24 of the 250 loci for PCR verification in four Caniformia species. All 24 loci were confirmed to be putative panda-specific (PPS) insertions that were absent in other species (four example loci are shown in Fig. 4) (Additional file 3).

To determine if these PPS elements were new insertion events, we examined the TSD sites and nucleotide divergence of these elements. Elements from an active subfamily should have intact TSDs at the insertion sites. Evident TSD sequences were observed in 78 of 139 PPS_SINEC1_AMe elements (56.1%), but only eight of 111 PPS_SINEC1B_AMe elements (7.0%) (Additional file 2).

**Fig. 5:**
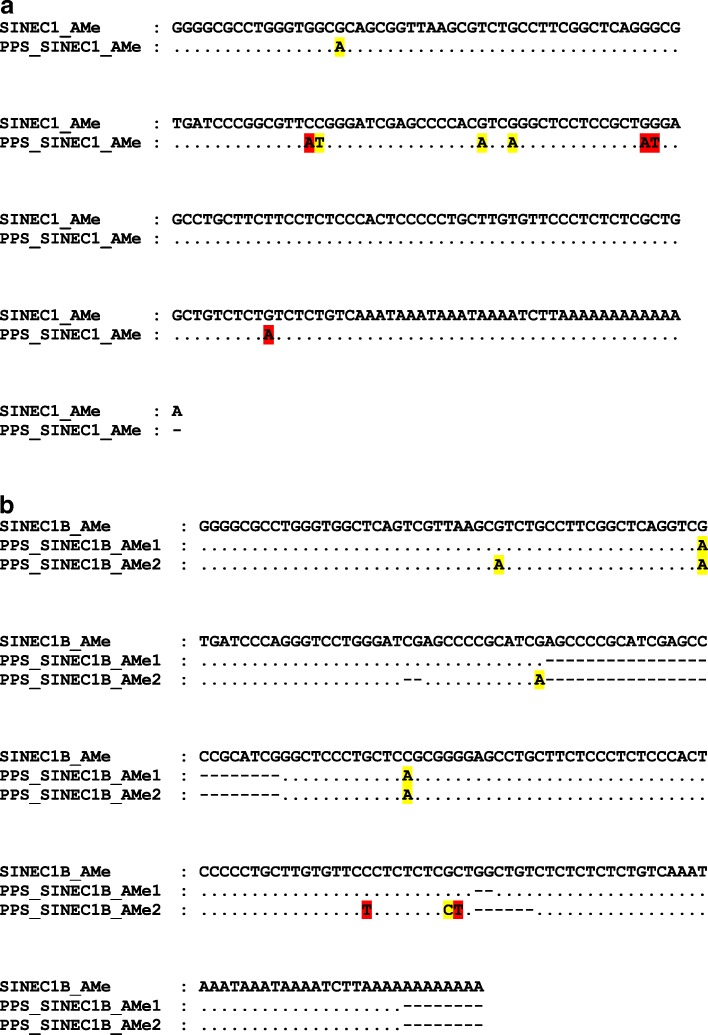
Alignments of PPS consensus sequences with RepBase consensus sequences. **a** SINEC1_AMe and PPS_SINEC1_AMe; **b** SINEC1B_AMe and PPS_SINEC1B_AMe. Dots represent the same nucleotides as the consensus sequence. Deletions are shown as dashes and mutations are shown as the correct base in different colors (yellow for CpG sites and red for non-CpG sites) for each sequence

**Fig. 6:**
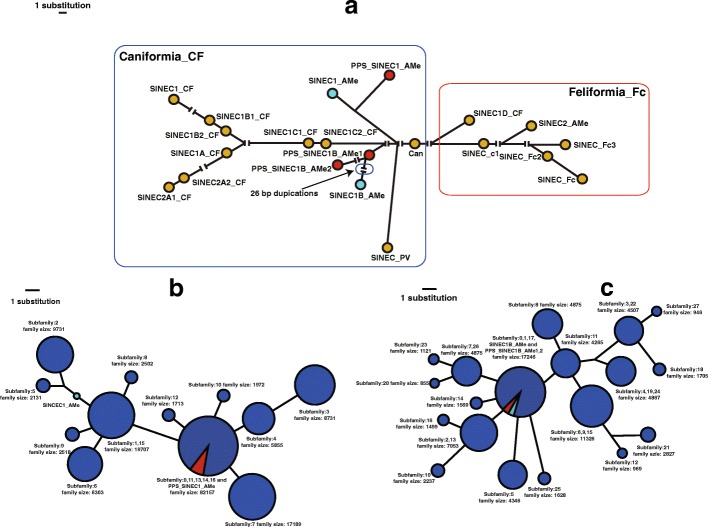
Median-joining network analysis. **a** Major Can_SINE consensus sequences from Repbase and COSEG PPS consensuses. The consensus sequences of PPS_SINEC1_AMe and PPS_SINEC1B_AMe generated by COSEG are displayed as red circles. The consensus sequences of SINEC1_AMe and SINEC1B_AMe from Repbase are displayed as cyan circles. Other Repbase Can_SINE consensus sequences are displayed as yellow circles. There are 26 duplications belong to SINEC1B_AMe consensus sequence in Repbase when compared with the PPS_SINEC1B_AMe. The ‘||’ represents gaps more than 1 bp. The two main clades, Caniformia_CF and Feliformia_Fc, are indicated by blue and red circle, respectively. **b** COSEG consensuses of SINEC1_AMe subfamilies and Repbase SINEC1_AMe consensus (cyan). **c** COSEG consensuses of SINEC1B_AMe subfamilies and Repbase SINEC1B_AMe (cyan). All blue circles represent consensus sequences reconstructed by COSEG in the panda genome. Circle size represents the subfamily size except the Repbase consensuses

To determine whether PPS elements were from potential new Can-SINE subfamilies, we conducted the COSEG to identify subfamily consensus among the 250 PPS elements. After removing elements with truncations and deletions (13 PPS_SINEC1_AMes), COSEG analysis generated one consensus from 126 PPS_SINEC1_AMe elements and two consensuses (PPS_SINEC1B_AMe1 and PPS_SINEC1B_AMe2) from 111 PPS_SINEC1B_AMe elements. Compared to the Repbase SINEC1_AMe consensus, PPS_SINEC1_AMe had eight point mutations, with four of them at CpG sites (Fig. 5a). PPS_SINEC1B_AMe1 consensus contains two CpG site mutations and two deletions, and PPS_SINEC1B_AMe2 consensus contains seven mutations (five at CpG sites) and three deletions compared to the Repbase SINEC1B_AMe (Fig. 5b). Next we used the Network analysis to examine the relationship between the new PPS consensuses and the known Can-SINE consensuses. Network analysis confirmed that PPS_SINEC1_AMe consensus had the closest relationship with SINEC1_AMe, and the two PPS_SINEC1B_AMe consensuses had close relationship with SINEC1B_AMe (Fig. 6a).

Compared to Repbase consensus sequences, the average divergence rates were 7.38 ± 1.37% for PPS_SINEC1_AMe elements, 11.86 ± 2.20% for PPS_SINEC1B_AMe1, and 12.60 ± 2.03% for PPS_SINEC1B_AMe2 (Additional file 2). These rates were smaller than the average divergences of original subfamilies (SINEC1_AMe, 8.82% and SINEC1B_AMe, 13.09%) indicating that the identified PPS elements are young and newly integrated elements in the panda genome. However, compared to the COSEG generated PPS consensus sequences, the divergence rates were 6.39 ± 3.27% for PPS_SINEC1_AMe elements, 14.30 ± 3.61% for PPS_SINEC1B_AMe1 and 16.53 ± 3.85% for PPS_SINEC1B_AMe2(Additional file 2). The average age of PPS_SINEC1_AMe was estimated to be ~ 15.20 Mya (based on BEAST) or ~ 17.42 Mya (based on CpG and non-CpG mutations), which was about the time the panda and polar bear diverged from each other. Additionally, estimated average age of PPS_SINEC1B_AMe1 was ~ 21.54 Mya (based on BEAST) and~ 31.83 Mya (based on CpG and non-CpG mutations). The average age of PPS_SINEC1B_AMe2 was estimated to be ~ 21.45 Mya and ~ 59.13 Mya (based on CpG and non-CpG mutations). It seems that these two SINEC1B_AMe subfamilies are relatively old.

To determine the possible functional impact of PPS elements, we compared their genomic locations to known genes in the panda genome. A total of 45 PPS_SINEC1_AMe, 29 PPS_SINEC1B_AMe1 and 13 PPS_SINEC1B_AMe2 elements inserted into genic regions. Only one PPS_SINEC1_AMe element inserted into a sequence of a non-coding RNA gene (*LOC105241841*), and all others inserted into intronic regions (Additional file 2).

### Potential new Can-SINE subfamily identification

Because SINEC1_AMe and SINEC1B_AMe are large Can-SINE subfamilies, they could contain smaller unique subfamilies, similar to *Alu* subfamilies in primate genomes [16]. Using COSEG analysis, we further examined the evolutionary dynamics of SINEC1_AMe and SINEC1B_AMe subfamilies to determine whether the three PPS consensuses were new clades within these subfamilies. COSEG analysis on all SINEC1_AMe elements in the panda genome divided SINEC1_AMe into 17 subfamilies, and SINEC1B_AMe elements into 28 subfamilies. These subfamilies were selected for Network analysis with three PPS consensuses and Repbase consensus. After excluding CpG sites, PPS_SINEC1_AMe was grouped with five SINEC1_AMe COSEG consensuses and formed a major group containing 82,157 elements (Fig. 6b). This group is the largest group and 4 substitutions away from the Repbase SINEC1_AMe consensus. By contrast, both PPS_SINEC1B_AMe1 and PPS_SINEC1B_AMe2 were grouped with the Repbase SINEC1B_AMe consensus and three other COSEG consensuses to form the largest group in the network, containing 17,246 elements (Fig. 6c).

Overall, PPS_SINEC1_AMe represents a major group of SINEC1_AMe element, the group has a relatively low divergence rate and young age, and its consensus contains several distinct diagnostic mutations compared to the SINEC1_AMe consensus. Therefore, we conclude that PPS_SINEC1_AMe elements represent a novel Can-SINE subfamily, that we named SINEC1_AMe2, a subfamily separating from the current SINEC1_AMe, following the TE subfamily naming convention [11].While, evidence from the two PPS_SINEC1B_AMe subfamilies was not sufficiently robust to be new Can-SINE subfamilies.

### Characteristics of the new Can-SINE subfamily

We then repeated RepeatMasker analysis against the panda and polar bear genome including the SINEC1_AMe2 consensus to characterize this new Can-SINE subfamily. A total of 143,166 and 106,889 SINEC1_AMe2 elements were found in the panda and the polar bear genomes, respectively. At least 96.8% of them (124,118 elements in the panda and 104,892 in the polar bear) have divergence rate of ≤10%, indicating they are young in both genomes. The SINEC1_AMe2 elements account for ~ 57% of SINE elements with divergence rates of ≤10% in the panda genome (Additional file 1: Table S3). In addition, we identified 19 SINEC1_AMe2 elements with a zero divergence rate in the panda genome, with all being panda specific loci and are absent at the orthologous regions of the polar bear genome. Similarly, we identified 49 SINEC1_AMe2 elements with a zero divergence rate in the polar bear genome that are polar bear specific loci. These results indicate that SINEC1_AMe2 is one of the youngest and most active SINE subfamily in the panda and polar bear genomes.

## Discussion

### Characteristics of Can-SINEs in the four genomes

We characterize TEs in the genomes of four Caniformia species, focusing on Can-SINEs. Despite similarities in the four major TE types, obvious differences exist among the four Caniformia genomes particularly in the young and active Can-SINEs. SINEC1_AMe and SINEC1B_AMe are the most abundant Can-SINE subfamilies in the panda and polar bear genomes, with relatively low divergence rates. In contrast, the number of elements in these two subfamilies is much lower in the dog genome. Instead, several other Can-SINE subfamilies (e.g., SINEC1C1_CF, SINEC2A1_CF) are the most abundant subfamilies in the dog. By contrast, the ferret genome accumulates the highest numbers of MVB2 in the four genomes. This extensive genomic variability will be a useful resource for the study of ancestral relationships among different Caniformia lineages. It is noted that some of the differences between genomes might be underestimated because the draft genomes of panda, polar bear, and ferret were assembled by short read sequences, which is known to lead the underestimation of some longer repeat sequence copy numbers [4, 29].

### The transposition activity of Can-SINEs

Previous studies have suggested that a retrotransposon subfamily accumulate its respective copies for a certain period of time and then become quiescent. Other newer subfamilies subsequently become active, and the pattern repeats itself [16, 30]. This pattern is well illustrated by the *Alu* family of SINEs in primates. During the early stages of primate evolution, *Alu*J subfamilies were active. The activity of these subfamilies was later reduced, and the *Alu*S subfamilies became active. The *Alu*Y subfamilies were even more taxonomically specific in that they began their expansion in primates more recently [16, 30]. The propagation of retrotransposon subfamilies in different lineages varies greatly over the evolution of Carnivora. Walters-Conte et al. [31] identified two Can-SINE subfamilies within the Feliformia (cat-like carnivores) suborder. These two subfamilies shared close relationships with the Repbase SINEC_Fc1 and SINEC_Fc2 subfamilies, and one of the two families (SINEC_Fc1) arose recently and showed evidence for active proliferation. Within the Caniformia, Wei and Kirkness [32] suggested that the SINEC_Cf repeats comprised a major Can-SINEs subfamily that had undergone recent expansion and identified at least 10,000 polymorphic SINEC_Cf loci in different dog breeds. These studies demonstrated that the transposition activity of Can-SINE subfamilies vary widely over evolutionary time, with periods of low and high activity. Moreover, the transposition rate varies greatly among different Caniformia lineages. Only a few Can-SINE subfamilies in each species demonstrate average divergence rates of ≤10% (Fig. 2b), indicating the young and active subfamilies are few in Caniformia as is found in other mammals [22].

TinT analysis indicated that SINEC1_AMe and SINEC1B_AMe subfamilies are the two most active subfamilies in the panda and polar bear genomes and have maintained high transposition activities for a long time (Fig. 3), which is further supported by their accumulated high copy numbers and relatively low divergence rates (Fig. 2). It is also worth noting that the transposition activity estimated by TinT might result from the lack of annotation of Can-SINE subfamilies in the panda and polar bear. It is possible that mutliple recent Can-SINE subfamilies are lumped under one big subfamily (such as SINEC1_AMe and SINEC1B_AMe), leading to an extended transposition activity period.

Unlike the panda and polar bear genomes, the two most active subfamilies in the dog genome are SINEC2A1_CF and SINEC1C1_CF, and these subfamilies have maintained transposition activity to the present in the TinT analysis. However, previous dog genome research [33] demonstrated SINEC_Cf was the dominant young subfamily probably because these two subfamilies have not been recognized until recently. In particular, the SINEC2A1_CF subfamily accumulates high copies (~ 180,000 elements) in the dog genome compared to the other three Caniformia species (only 11–103 elements) (Fig. 2a). It also has the lowest average divergence rate (5.77%, Fig. 2b) and the longest period of transposition activity (Fig. 3). All the results suggest that the SINEC2A1_CF subfamily has actively propagated specifically in the dog lineage and may have played an important role in shaping the architecture of the dog genome. We also found that MVB2 is the dominant Can-SINE subfamily in the ferret genome with the highest number, relatively low divergence rate and the longest period of transposition activity. Overall, it is suggested that different Can-SINE subfamilies actively propagated during the divergence and radiation of Caniformia lineages, represent a great source of genomic diversity among Caniformia species.

The differences in Can-SINE amplification between the four genomes might due to the characteristics of SINE amplification. During evolution, only a few SINE insertions function as source of novel SINE transcripts during amplification at any given time [34]. With the accumulation of mutations, transcription of a given master copy will eventually be inhibited and be replaced by an alternate copy. As results, SINE subfamilies possess the diagnostic nucleotide sequence to be classified into phylogenetic lineages [35]. Our results of Can-SINE amplification are in line with the evolutionary relationships between the four Caniformia species. The dog is an outlier group from the other genomes, and has the most varied Can-SINE subfamilies, in particular the Can-SINE “CF” subfamilies. Similarly, the ferret genome accumulated the highest numbers of MVB2 probably due to the closer relationship of ferret to the America mink than other species.

Considering a large number (10,000) of polymorphic SINE_Cf in dogs [32], the differences of Can-SINE transposition activity in the four genomes might be underestimated because these polymorphic loci could not be detected in one reference genome and thus this was not investigated in the present study. Besides, polymorphic loci in the four Caniformia species might lead to ambiguous results for identification of species-specific Can-SINE elements. Further studies with genomes from more individuals will assist in identifying the effects of polymorphic elements.

### The characteristics of putative panda-specific Can-SINEs and the new Can-SINE subfamily

After the panda-associated genome alignments, we identified 250 PPS elements in the panda genomes that are absent from the orthologous regions in the other three genomes. Further investigation of these PPS insertions allowed us to identify a potentially new Can-SINE subfamily, SINEC1_AMe2, which is distinguished from the current SINEC1_AMe subfamily. SINEC1_AMe2 has similar structures to other Can-SINEs with a conservative tRNA-related region [36]. However, its consensus sequence is distinguishable from the SINEC1_AMe consensus by eight mutations with half of the mutations outside of the hypervariable CpG sites. In the Network analysis of COSEG consensuses within SINEC1_AMe, SINEC1_AMe2 formed the group with the highest copy number. Additionally, several lines of evidence suggest that it is generated from a source element different from SINEC1_AMe. First, the SINEC1_AMe2 consensus was constructed from the panda-specific elements that are young and intact elements with apparent TSD sequences and relatively long poly-A/T tail. Second, SINEC1_AMe2 subfamily had more than 100,000 copies in the panda and polar bear genomes, suggesting it is a major subfamily with high activity. Third, the majority of SINEC1_AMe2 had divergence rates of ≤10%, suggesting it is a younger subfamily than SINEC1_AMe and it represents most of the young elements within the current SINEC1_AMe subfamily (Additional file 1: Table S3). In particular, we identified 19 and 49 SINEC1_AMe2 elements in the panda and polar bear genome with a zero divergence rate. In contrast, no such elements were found in SINEC1_AMe elements, indicating SINEC1_AMe2 is different from SINEC1_AMe. All of these 68 elements are panda or polar bear specific loci, which provides a strong support that the SINEC1_AMe2 subfamily is still active in the panda and polar bear genomes. Considering all of the evidence, we conclude that SINEC1_AMe2 is a new subfamily separate from SINEC1_AMe, and it started integrating into the genomes of the panda and polar bear 15–17 Mya. Since then it has been active and successfully propagated as the dominant active subfamily in both genomes.

By contrast, the data does not support the PPS_SINEC1B_AMe elements form a new Can-SINE subfamily. Although two consensuses were generated from young PPS_SINEC1B_AMe elements, they are shorter than SINEC1B_AMe consensus, and have most of mutations at CpG sites or (CT)_n_ sites which might not be fixed diagnostic mutations. Furthermore, elements in these subfamilies lack TSD sequences, exhibit relatively high divergence rates, and the estimated insertion age is old. The two PPS_SINEC1B_AMe consensuses were grouped with SINEC1B_AMe consensus in the network analysis (Fig. 6c), which indicates they are similar to SINEC1B_AMe consensus. In combination, the evidence does not support that PPS_SINEC1B_AMe elements are from a new subfamily within SINEC1B_AMe. It is worth noting that not all new Can-SINE subfamilies have been surveyed in the present study, some clades of subfamilies in the SINEC1_AMe and SINEC1B_AMe Network might contain smaller new subfamilies that are worthy of more comprehensive investigations in the future.

## Conclusions

In the present study, we reannotated TEs based on updated Repbase library and conducted a systematical comparison of Can-SINEs compositions, subfamilies and transposition activities between the genomes of four Caniformia species: panda, polar bear, dog, and ferret. There are significant differences in the copy numbers, average divergence rates and transposition activities among Can-SINE subfamilies in the four genomes. We identified 139 PPS_SINEC1_AMe elements and 111 PPS_SINEC1B_AMe elements that inserted in the panda genome but were absent at the orthologous regions of the other three genomes. Further investigations of these PPS insertions allowed us to identify a new Can-SINE subfamily, SINEC1_AMe2, which is distinguishable from the current SINEC1_AMe subfamily. Combing evidences from different analysis, we conclude that SINEC1_AMe2 is a young subfamily and has been active and successfully propagated to be the dominant active subfamily in the panda and polar bear genome.

## Methods

### TE annotation of the four Caniformia genomes

The genome assemblies of four Caniformia species (AilMel_1.0, CanFam3.1, MusPutFur1.0, and UrsMar_1.0) were downloaded from NCBI [37]. The scaffold N50 for each genome assembly varied from 1,281,781-15,940,661 bp (Additional file 4). The genome assemblies were analyzed for TE composition using a local installation of the RepeatMasker program [38]. The standard Repbase consensus library (version 2015.8.7) was used for the analysis [39]. Cross_Match v0.990329 [40] was used for sequence search in the RepeatMasker program. Perl-scripts calcDivergence-fromAlign.pl and RepeatLandscape.pl in the RepeatMasker package were used to create the repeat landscape.

Using RepeatMasker annotation, the frequencies, average divergence rates, proportion of genome, and subfamily distributions of all TEs in the four genomes were extracted and calculated using a custom Perl script and R statistic software (version 3.2.3).

### Transposition in transposition analysis

TinT analysis can be used to determine the relative age of transposon families [27, 28], and has been successfully applied in primates, sharks, and amniotes transposons studies [41–44]. TinT analyses were performed for Can-SINEs in panda, polar bear, ferret and dog genomes with default parameters, except for Minimum Repeat Extension (4).

### Panda-associated two-way genome alignments

We performed in silico screening for putative panda-specific elements based on the two-way alignments described in Kent et al. [45] and Doronina et al. [46]. All elements belonging to the two most active SINE subfamilies in the panda genome (SINEC1_AMe and SINEC1B_AMe) were selected. Elements in these subfamilies were filtered for the length (SINEC1_AMes ≥150 bp, SINEC1B_AMes ≥170 bp) and the average divergence rate (SINEC1_AMes ≤10%, SINEC1B_AMes ≤15%) to obtain potential young insertions. The selected elements were extracted with 500 bp of flanking sequence on each side for comparison of orthologous loci among the four Caniformia species. A local installed Blast+ 2.2.28 tool was used to compare the flanking sequences to other three genomes (panda vs polar bear/dog/ferret) [47]. The orthologous loci of these elements in the four species were then obtained. Loci with alignment length covering ≥90% of the query sequence and having identity ≥80% were selected. A locus that had ≥80% identity in the flanking sequence across all species was considered an orthologous locus and is referred as “orthologous locus” in the text. A custom Perl script (parse_blast6.out.pl) was then used to convert the results to the gff format. Bedtools [48] was used to determine the overlapped region between the Blast+ and RepeatMasker outputs. Elements that were present in the panda genome and absent in the other three genomes at the orthologous loci were considered PPS elements. To investigate distributions of these PPS elements, the annotation file for the panda genome was downloaded from NCBI [49] and Bedtools was used to obtain the overlapping regions between PPS elements and genes.

### Analyses of PPS Can-SINE elements

The COSEG program [50] has been designed to identify repeat subfamilies based on significant co-segregating mutations. During analysis, COSEG ignores non-diagnostic mutations and potentially misleading mutational events to give an accurate representation of relationships between subfamilies of elements. We applied COSEG program to PPS_SINEC1_AMe and PPS_SINEC1B_AMe elements to generate the consensus sequences for subfamilies. The minimum subfamily size of COSEG was set at 10 for PPS elements. The Repbase SINEC1_AMe and SINEC1B_AMe consensus sequences [39] were selected as original template for PPS_SINEC1_AMe and PPS_SINEC1B_AMe respectively. To better understand the relationships between the PPS subfamilies and other Can-SINE subfamilies, Network analysis was then performed combining consensus of PPS_SINEC1_AMe, and PPS_SINEC1B_AMe with consensus sequences of all Can-SINE subfamilies downloaded from Repbase [39, 51]. All consensus sequences were aligned using Mega5 software [52] and converted to the .rdf file format by the DNAsp program [53]. The .rdf files were then imported to the Network program, and the median-joining analyses were run.

We also performed COSEG analysis on the all SINEC1_AMes elements and SINEC1B_AMes elements in the panda genome separately to investigate potential subfamilies within them. The minimum subfamily size of COSEG was set at 1623 for SINEC1_AMes and 803 for SINEC1B_AMes, and the original template was their corresponding Repbase consensus sequence (SINEC1_AMe and SINEC1B_AMe, respectively). Then the Network analysis was conducted on all COSEG SINEC1_AMe consensuses plus PPS_SINEC1_AMe, and all SINEC1B_AMe consensus plus PPS_SINEC1B_AMe with Repbase consensus, to investigate relationships of the PPS subfamilies to other SINEC1_AMe and SINEC1B_AMe elements.

We manually checked the target site duplication sequences for all identified PPS elements. To do this, 30 bp flanking sequence including TSDs was extracted by an in-house Perl script. The 6–15 bp identical flanking sequences or flanking sequences with one different nucleotide on both sides of an element were regarded as the TSDs.

### Age estimation of PPS_SINEC1_AMe subfamily

Two methods were applied to estimate the age of PPS SINEC1 subfamilies. For the first method, the consensus sequences of PPS_SINEC1_AMe and PPS_SINEC1B_AMe were aligned with their members using Mega5 software after removing insertions and poly (A/T) tails. Elements that contained deletions larger than 50 bp were excluded from the analysis. Due to the low-quality alignments in the (CT)n regions, the region was also excluded from the age estimates of the PPS_SINEC1B_AMe elements. Mutations of both subfamilies were divided to CpG and non-CpG mutations. An age estimate for each subfamily was determined by an average of results based on the two types of mutations (CpG and non-CpG mutations) with a custom Perl script [54]. For the second method, the BEAST program, applied to estimate dates of divergence using transposon data previously published [55, 56], was used to estimate the age of the PPS_SINEC1_AMes and PPS_SINEC1B_AMes elements with the following parameters: Site Heterogeneity = ‘gamma’; Clock = ‘strict clock’; Species Tree Prior = ‘Yule Process’; Prior for tmrca = ‘Normal distribution’ with 2.0 standard deviation; Prior for clock.rate = ‘Uniform’ with initial rate set at 0.0013 (neutral mutation rate) and upper rate set at 0.0104 (CpG mutation rate) to include the major mutation rates found in the panda genome. All other parameters were set at default settings. All chosen parameters are similar to the previous study [57]. The estimated age was determined by tmrca with a calibration time of 15.5 Mya based on the divergence time between the panda and polar bear reported in previous studies (12~ 19 Mya) [4, 21, 23, 58, 59].

### PCR validation and DNA sequencing of PPS can-SINEs

To validate the PPS elements, PCR was performed on a four-species Caniformia DNA panel including the giant panda, polar bear, dog and mountain weasel (*Mustela altaica*) (a species closely related to the ferret) (Additional file 5). Twenty-three SINEC1_AMes and one SINEC1B_AMe were randomly chosen for validation. Primers were designed based on the 300 bp flanking sequences using Primer 3 [60, 61] and were listed in Additional file 3. PCR amplification on each locus was performed in 20 μL reactions with 10–50 ng genomic DNA, 60 nM of each oligonucleotide primer, 10 μL Master Mix. PCR reaction conditions were as follows: an initial denaturation at 94 °C for 5 min, followed by 35 cycles of denaturation at 94 °C, annealing at the previously determined optimal annealing temperature (Additional file 3), and extension at 72 °C for 1 min each, followed by a final extension of 72 °C for 5 min. PCR products were analyzed on 2% agarose gels stained with 0.5 μL GoldView (TIANGEN) and visualized with UV fluorescence. Additionally, all PCR products were Sanger sequenced to verify bp composition [62].

### Characteristics of the potential new subfamily

We rerun the RepeatMasker program against the giant panda and polar bear genomes by combining the consensus of the potential new SINEC1_AMe2 subfamily with other Repbase Can-SINE consensuses. Elements belonging to the potential new subfamily were described, from where elements with zero divergence rate to the consensus were identified. The 500 bp flanking sequences on both sides of these young elements were further extracted to investigate the presence/absence pattern in the panda and polar bear genomes.

## Additional files

Additional file 1: **Table S1.** TEs with divergence rate <=10% in the four carnivore genomes. **Figure S1.** Divergence rate distribution of four major types of TEs in panda (a), polar bear (b), dog (c), and ferret (d) genomes. **Table S2.** SINE subfamilies with average divergence rate ≤10% in the panda genome. **Table S3.** SINE (including SINEC1_AMe2) subfamilies with average divergence rates ≤10% in the panda genome. (DOCX 25 kb)

Additional file 2: The PPS_SINEC1_AMe and PPS_SINEC1B_AMe elements’ information. (XLSX 61 kb)

Additional file 3: The primers used for PPS SINE validation and the orthologous statistics. (XLSX 11 kb)

Additional file 4: The genomes information. (XLSX 10 kb)

Additional file 5: The sample information for the PPS SINE PCR validation panel. (XLSX 9 kb)

## Abbreviations

ERV: Endogenous retroviruse
GP: Genome percentage
LINE: Long interspersed element
LTR: Long terminal repeat
Mya: Million years ago
PPS: Putative panda-specific
SINE: Short interspersed element
TE: Transposable element
TinT: Transposition in transposition
tmrca: Time of most recent common ancestor
TSD: Target site duplication
