# Supplementary material for: Can-SINE dynamics in the giant panda and three other Caniformia genomes
Source: Mob DNA. 2018 Nov 10;9:32. doi: 10.1186/s13100-018-0137-0 (PMC6230240; doi:10.1186/s13100-018-0137-0)
Supplement: Supplementary file 1 — Table S1. TEs with divergence rate <=10% in the four carnivore genomes. Figure S1. Divergence rate distribution of four major types of TEs in panda (a), polar bear (b), dog (c), and ferret (d) genomes. Table S2. SINE subfamilies with average divergence rate ≤10% in the panda genome. Table S3. SINE (including SINEC1_AMe2) subfamilies with average divergence rates ≤10% in the panda genome. (DOCX 25 kb) [file 13100_2018_137_MOESM1_ESM.docx]

**Table S1 TEs with divergence rate <=10% in the four carnivore genomes**

|  | **Panda** |  | **Polar bear** |  | **Dog** | |  | **Ferret** | |  |
| --- | --- | --- | --- | --- | --- | --- | --- | --- | --- | --- |
| **Types** | **Counts** | **Gp^*^** | **Counts** | **Gp** | **Counts** | **Gp** | | **Counts** | **Gp** | |
| **SINE** | **226,459** | **1.83%** | **222,343** | **1.81%** | **392,978** | **2.79%** | | **214,254** | **1.54%** | |
| **LINE** | **50,453** | **1.40%** | **43,262** | **1.18%** | **59,301** | **2.25%** | | **11,290** | **0.07%** | |
| **LTR** | **3,892** | **0.06%** | **4,121** | **0.07%** | **4,208** | **0.11%** | | **1,406** | **0.01%** | |
| **DNA** | **1,260** | **0.00%** | **1,236** | **0.00%** | **954** | **0.00%** | | **816** | **0.00%** | |

***Gp: Genome percentage**

**Figure S1 Divergence rate distribution of four major types of TEs in panda (a), polar bear (b), dog (c), and ferret (d) genomes.** The divergence rate was calculated between their identified TE elements in the genome and the consensus sequences (Repbase).

**
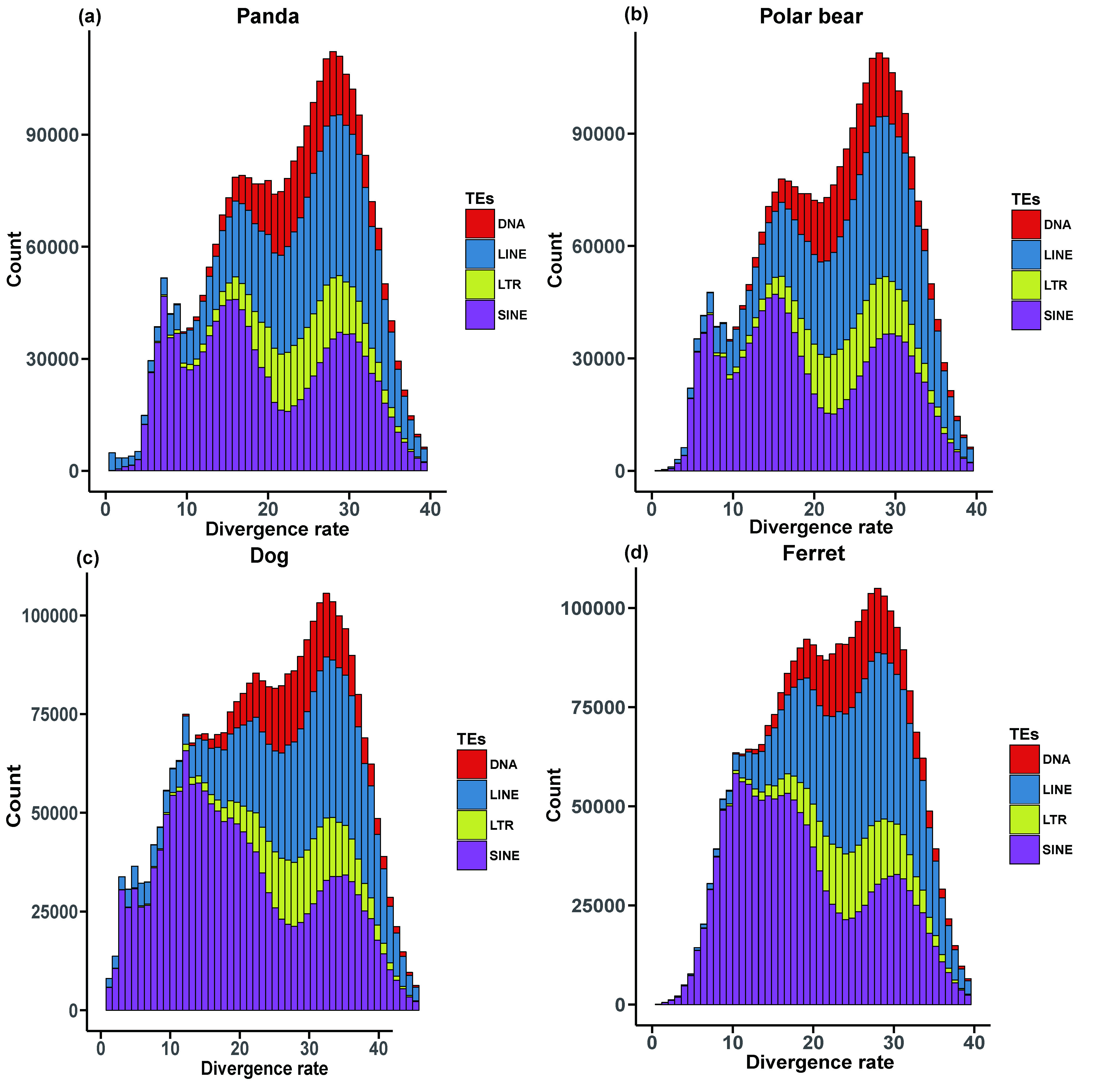
**

**Table S2 SINE subfamilies with average divergence rate ≤10% in the panda genome**

| **SINE Type** | **Length (bp)** | **Percentage** | **Copy number** | **Copy number Percentage** |
| --- | --- | --- | --- | --- |
| SINEC1_AMe | 34,596,819 | 82.93% | 180,010 | 79.49% |
| SINEC1B_AMe | 4,875,793 | 11.69% | 27,122 | 11.98% |
| SINEC_b2 | 560,175 | 1.34% | 4,867 | 2.15% |
| SINEC_b1 | 468,170 | 1.12% | 3,747 | 1.65% |
| tSINE_Fc | 393,906 | 0.94% | 3,060 | 1.35% |
| SINEC2_AMe | 246,012 | 0.59% | 2,068 | 0.91% |
| CAN | 133,085 | 0.32% | 744 | 0.33% |
| SINEC_c1 | 127,222 | 0.30% | 796 | 0.35% |
| SINEC_c2 | 109,892 | 0.26% | 1,079 | 0.48% |
| SINEC_old | 67,204 | 0.16% | 760 | 0.34% |
| SINEC1C2_CF | 28,760 | 0.07% | 187 | 0.08% |
| SINEC_Fc3 | 20,108 | 0.05% | 410 | 0.18% |
| SINEC_Mv | 18,987 | 0.05% | 433 | 0.19% |
| SINEC_a1 | 15,522 | 0.04% | 59 | 0.03% |
| SINEC1C1_CF | 13,004 | 0.03% | 89 | 0.04% |
| MIRc | 6,442 | 0.02% | 177 | 0.08% |
| MVB2 | 5,843 | 0.01% | 33 | 0.01% |
| SINEC_Pv | 5,237 | 0.01% | 105 | 0.05% |
| MIR | 4,821 | 0.01% | 129 | 0.06% |
| MIR3 | 4,570 | 0.01% | 127 | 0.06% |
| SINEC_a2 | 4,520 | 0.01% | 59 | 0.03% |
| MIR1_Amn | 2,813 | 0.01% | 85 | 0.04% |
| MIRb | 2,569 | 0.01% | 69 | 0.03% |
| SINEC_Fc2 | 2,079 | 0.00% | 32 | 0.01% |
| SINEC_Cf2 | 1,651 | 0.00% | 27 | 0.01% |
| SINEC_Cf3 | 1,141 | 0.00% | 16 | 0.01% |
| SINEC_Fc | 665 | 0.00% | 9 | 0.00% |
| MamSINE | 337 | 0.00% | 7 | 0.00% |
| AmnSINE | 317 | 0.00% | 9 | 0.00% |
| SINE_FS | 144 | 0.00% | 4 | 0.00% |
| SINEC_Cf | 140 | 0.00% | 2 | 0.00% |
| LFSINE_Vert | 121 | 0.00% | 4 | 0.00% |
| SINE_OM | 72 | 0.00% | 1 | 0.00% |
| SINE_FN | 28 | 0.00% | 1 | 0.00% |

**Table S3 SINE (including SINEC1_AMe2) subfamilies with average divergence rates ≤10% in the panda genome**

| **SINE Type** | **Length(bp)** | **Percentage** | **Copy number** | **Copy number Percentage** |
| --- | --- | --- | --- | --- |
| SINEC1_AMe2 | 24,632,856 | 57.47% | 124,118 | 52.49% |
| SINEC1_AMe | 10,803,543 | 25.20% | 60,599 | 25.63% |
| SINEC1B_AMe | 5,140,363 | 11.99% | 28,583 | 12.09% |
| SINEC_c2 | 611,299 | 1.43% | 7,341 | 3.10% |
| SINEC_b2 | 572,543 | 1.34% | 5,175 | 2.20% |
| SINEC_b1 | 423,277 | 0.99% | 2,793 | 1.18% |
| SINEC2_AMe | 253,063 | 0.59% | 2,125 | 0.90% |
| SINEC_old | 145,145 | 0.34% | 1,618 | 0.68% |
| SINEC_c1 | 129,658 | 0.30% | 1,541 | 0.65% |
| SINEC_Fc3 | 60,676 | 0.14% | 859 | 0.36% |
| SINEC_Mv | 19,466 | 0.05% | 440 | 0.19% |
| SINEC_a1 | 17,868 | 0.04% | 214 | 0.09% |
| SINEC_Fc2 | 14,179 | 0.03% | 172 | 0.07% |
| MIRc | 6,442 | 0.02% | 177 | 0.07% |
| SINEC_Pv | 6,014 | 0.01% | 113 | 0.05% |
| SINEC_a2 | 5,009 | 0.01% | 64 | 0.03% |
| MIR | 4,731 | 0.01% | 127 | 0.05% |
| MIR3 | 4,570 | 0.01% | 127 | 0.05% |
| MIR1_Amn | 2,813 | 0.01% | 85 | 0.04% |
| MIRb | 2,569 | 0.01% | 69 | 0.03% |
| SINEC_Cf2 | 2,564 | 0.01% | 35 | 0.01% |
| SINE_OM | 2,080 | 0.00% | 31 | 0.01% |
| SINEC_Cf3 | 1,574 | 0.00% | 20 | 0.01% |
| SINEC_Fc | 1,482 | 0.00% | 20 | 0.01% |
| MamSINE1 | 337 | 0.00% | 7 | 0.00% |
| AmnSINE1 | 317 | 0.00% | 9 | 0.00% |
| SINEC_Cf | 195 | 0.00% | 3 | 0.00% |
| SINE_FS | 144 | 0.00% | 4 | 0.00% |
| LFSINE_Vert | 121 | 0.00% | 4 | 0.00% |
| SINE_FN | 28 | 0.00% | 1 | 0.00% |
